# Abdominal Distension in an Eleven-Month-Old Male

**DOI:** 10.29252/beat-080108

**Published:** 2020-01

**Authors:** Daniel Quesada, Larissa Morsky, Phillip Aguìñiga-Navarrete, Laura Castro, Luke Kim

**Affiliations:** 1 *Kern Medical, Bakersfield, CA; LAC + USC Medical Center, Los Angeles, CA, USA*; 2 *Kern Medical, Bakersfield, California, USA*

**Fig. 1:**
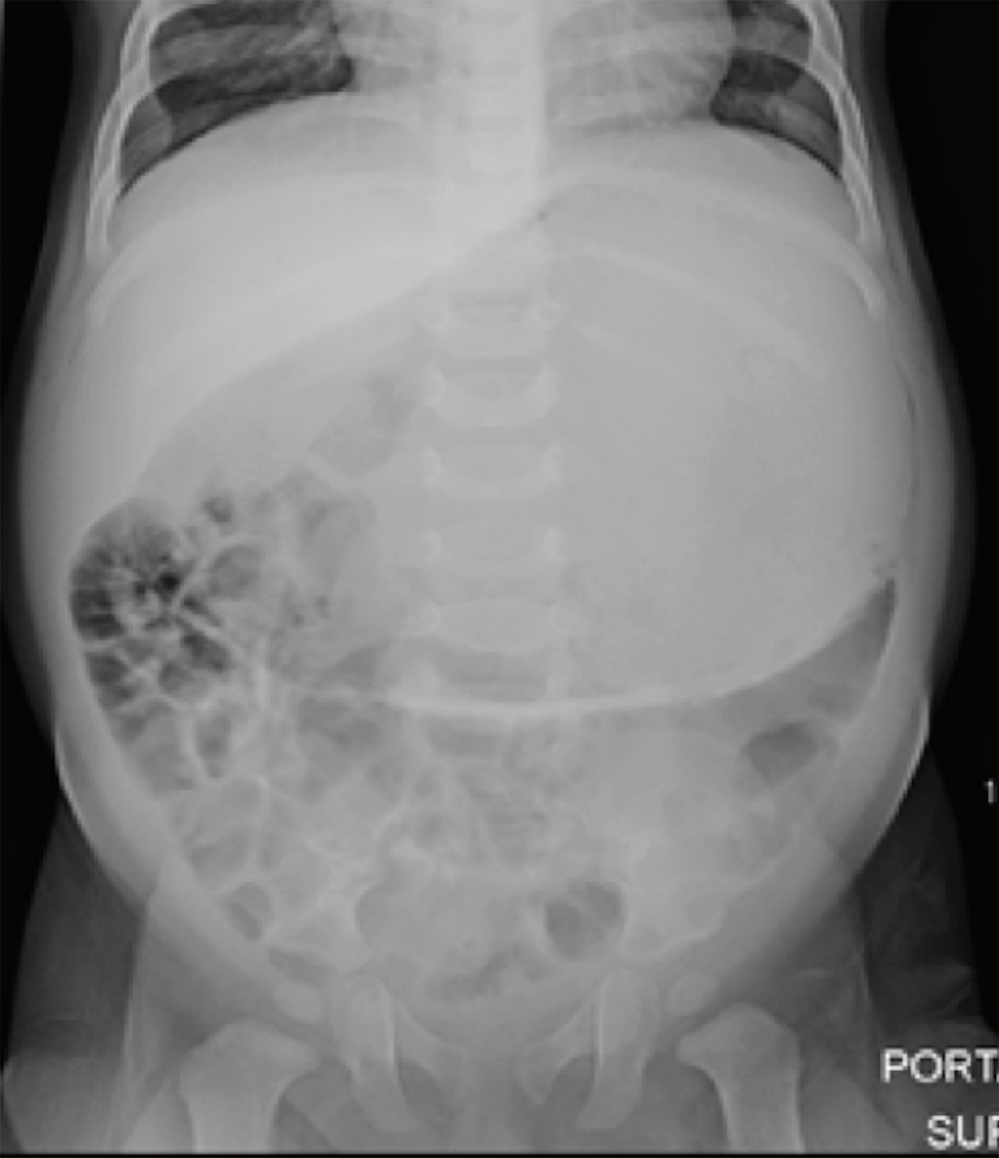
Kidney, ureter, and bladder (KUB) radiograph revealing severe gastric distension with normal bowel gas pattern concerning for gastric volvulus

An eleven-month-old male was brought in for a 1-day history of abdominal distension and anorexia associated with increased fussiness and multiple bouts of non-bloody diarrhea without any vomiting. A kidney, ureter and bladder (KUB) radiograph and abdominal ultrasound were obtained (Figure 1). 

**Fig. 2:**
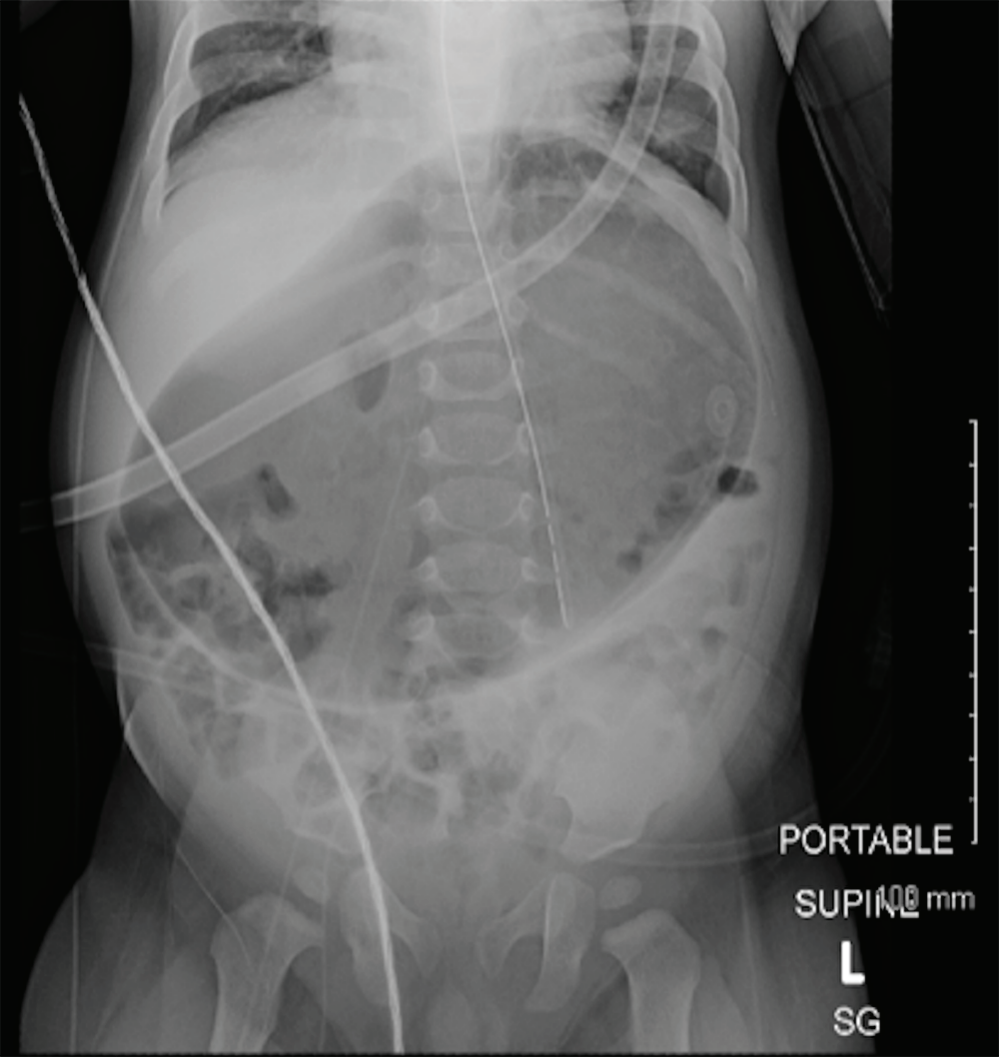
KUB with evidence of severe abdominal distension status post nasogastric tube placement (white arrow)

**Fig. 3:**
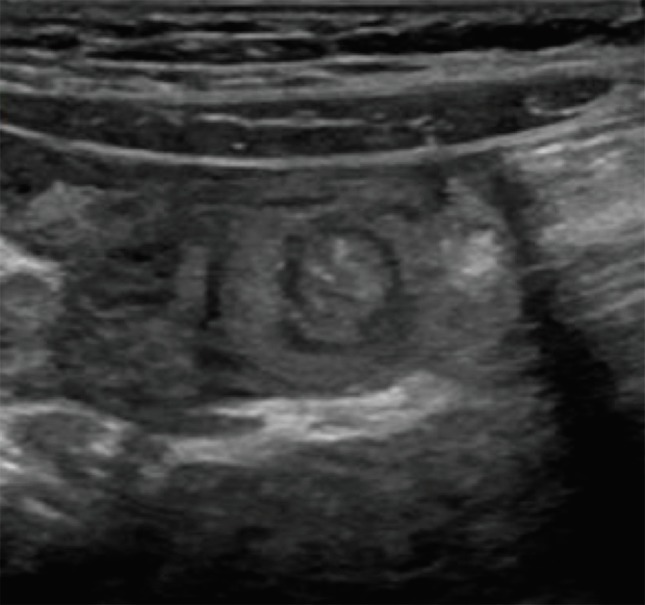
Abdominal ultrasound demonstrating the classic “target sign” of intestinal intussusception (white arrow)

After placing a nasogastric (NG) tube (Figure 2), a repeated KUB showed gastric decompression with the NG tube extending into the stomach. An abdominal ultrasound showed transient small bowel to small bowel intussusception in the left lower quadrant, which spontaneously decreased during the course of the examination (Figure 3). The upper gastrointestinal series-small bowel follows through (UGIS-SMFT) was reported negative for malrotation, but showed evidence of GERD. The patient was discharged the following day with a diagnosis of abdominal distension concerning for gastric volvulus with intermittent intussusception and moderate dehydration.

Both gastric volvulus and intussusception are life threatening conditions in pediatric patients that may present with a history of vomiting, abdominal pain and abdominal tenderness. However, given that symptoms are often nonspecific, a high index of suspicion must be maintained in ill-appearing children with abdominal complaints [1, 2]. When there is concern for gastric volvulus, acute diagnostic modalities include an acute abdominal film, chest x-ray, upper GI series with contrast, and abdominal ultrasound [3, 4]. A nasogastric tube should be placed to decompress the proximal obstruction created by the closed loop. Definitive treatment remains emergent surgical repair in the form of gastropexy [5]. Abdominal ultrasound and radiologists capable of performing air enema reductions under fluoroscopy are key factors for early management of intussusceptions [6].
